# MT1-MMP dependent repression of the tumor suppressor SPRY4 contributes to MT1-MMP driven melanoma cell motility

**DOI:** 10.18632/oncotarget.5258

**Published:** 2015-09-12

**Authors:** Khvaramze Shaverdashvili, Keman Zhang, Iman Osman, Kord Honda, Rauli Jobava, Barbara Bedogni

**Affiliations:** ^1^ From the Department of Biochemistry, Case Western Reserve University School of Medicine, Cleveland, OH, USA; ^2^ From the Departments of Dermatology, Urology and Medicine, New York University Langone Medical Center, New York, NY, USA; ^3^ From the Department of Pathology and Dermatology, Case Western Reserve University School of Medicine, Cleveland, OH, USA; ^4^ From the Department of Genetics, Case Western Reserve University School of Medicine, Cleveland, OH, USA

**Keywords:** cell migration, melanoma, SPRY4, matrix metalloproteinase (MMP)

## Abstract

Metastatic melanoma is the deadliest of all skin cancers. Despite progress in diagnostics and treatment of melanoma, the prognosis for metastatic patients remains poor. We previously showed that Membrane-type 1 Matrix Metalloproteinase (MT1-MMP) is one of the drivers of melanoma metastasis. Classically, MT1-MMP regulates a verity of cellular functions including cell-to-cell interaction and cell-to-matrix communication. Recently, MT1-MMP has been found to also modulate gene expression. To specifically assess MT1-MMP dependent gene regulation in melanoma, microarray gene expression analysis was performed in a melanoma cell line whose metastatic properties depend on the activity of MT1-MMP. We identified the tumor suppressor gene *SPRY4* as a new transcriptional target of MT1-MMP that is negatively regulated by the protease. Knockdown of MT1-MMP enhances SPRY4 expression at the mRNA and protein level. SPRY4 expression inversely correlates with that of MT1-MMP in melanoma samples and importantly, correlates with melanoma patient survival. SPRY4 modulates MT1-MMP dependent cell migration such that inhibition of SPRY4 rescues cell migration that has been impaired by MT1-MMP knock down. MT1-MMP decreases SPRY4 in part through an MMP2/RAC1 axis we previously show promotes cell motility downstream of MT1-MMP. These results identify the tumor suppressor SPRY4 as a novel molecular effector of MT1-MMP affecting melanoma cell motility.

## INTRODUCTION

Metastatic melanoma is an aggressive form of skin cancer, which is characterized by rapid progression, resistance to almost all current treatments and low survival rates in patients with metastatic disease [1]. Despite various therapeutic strategies for melanoma that have improved the life expectancy of patients, melanoma remains the deadliest of all skin cancers, mostly because of the high rate of tumor recurrence and distant metastasis after resection at late stages [2, 3]. The molecular mechanisms of melanoma metastasis development remain not well understood.

The invasion and spread of malignant cells involves reorganization and degradation of the extracellular matrix (ECM) through the activation of different proteolytic systems [4–8]. MT1-MMP is a zinc dependent type-1 transmembrane metalloproteinase that plays important roles in the regulation of ECM proteolysis and cellular migration in a number of cancers including malignant melanoma [6–13]. We have previously shown that MT1-MMP is upregulated during progression to invasive melanoma, whereas depletion of MT1-MMP significantly reduces melanoma metastasis [14].

Aside from its canonical function as a proteolytic enzyme and activator of a number of other MMPs and growth factors [15, 16], several groups have now shown that MT1-MMP can influence the transcription of tumor promoter genes. MT1-MMP promotes the expression of Vascular Growth Factor (VEGF), through phosphorylation and activation of Src kinase [17, 18]; and can move to the nucleus to trigger the expression and activation of a phosphoinositide 3-kinase δ (PI3Kδ)/Akt/GSK3β signaling cascade resulting in the modulation of macrophages immune responses [19].

To better understand the mechanisms underlying MT1-MMP dependent melanoma metastasis, we investigated the landscape of genes regulated by MT1-MMP specifically in melanoma cells. Among the genes identified, we focused on a class of tumor suppressor genes that resulted negatively regulated by MT1-MMP.

SPRY (SPROUTY) proteins are negative regulators of growth factor signaling pathways, hence their role as tumor suppressors; and their expression is altered in a number of human cancers [20]. In mammals there are four SPRY proteins: SPRY3, whose expression is detected only in brain and testes, and SPRY1, 2 and 4 which are ubiquitously expressed in embryonic and adult tissues [20, 21].

Growth factor receptor signaling pathways are important during embryonic development. SPRY proteins are intracellular negative regulators of such signaling molecules during neural crest cell formation, migration, differentiation and organogenesis [22, 23]. For instance overexpression of SPRY1 in Neural Crest Cells (NCC) results in inhibition of proliferation and increased apoptosis [24].

SPRY proteins are not fully complementary to each other, thus loss of just one of the SPRY genes may alter cell proliferative and tumorigenic properties [25–27]. For instance, in prostate and breast cancers SPRY1 and SPRY2 levels are inhibited, whereas in hepatocellular carcinoma only SPRY2 but not SPRY1 expression is decreased [28, 29]. SPRY1 mRNA and protein levels are increased in lung cancer tissue while SPRY2 and 4 levels are decreased and they function as tumor suppressors in these tumor [30]. On the other hand, SPRY2 is necessary for fibroblast transformation [31] and sarcoma formation and SPRY1 is important in rhabdomyosarcoma development [32]. These data suggest that SPRY proteins can have different functions depending on the tumor types.

In our study, we provide the first evidence that the tumor suppressor SPRY4 is a downstream target of MT1-MMP in melanoma. SPRY4 expression is diminished in metastatic melanoma compared to primary tumors. In addition, we found that higher levels of SPRY4 correlate with better prognosis and longer survival of melanoma patients. We show that an inverse relationship between MT1-MMP and SPRY4 exists such that overexpression of MT1-MMP downregulates the expression of SPRY4 and, vice versa, inhibition of MT1-MMP results in increased SPRY4. Finally, we demonstrate that inhibition of SPRY4 is sufficient to increase melanoma cell migration and is able to rescue cell motility that is impaired by the downregulation of MT1-MMP. Mechanistically, we find that MT1-MMP downregulates SPRY4 expression trough an MMP2/RAC1 axis we previously showed promotes melanoma cell motility downstream of MT1-MMP [14]. Taken together, these data suggest that MT1-MMP promotes melanoma metastasis in part by inhibiting the tumor suppressor SPRY4.

## RESULTS

### MT1-MMP regulates SPRY1, SPRY2, and SPRY4 transcripts

**Figure 1:**
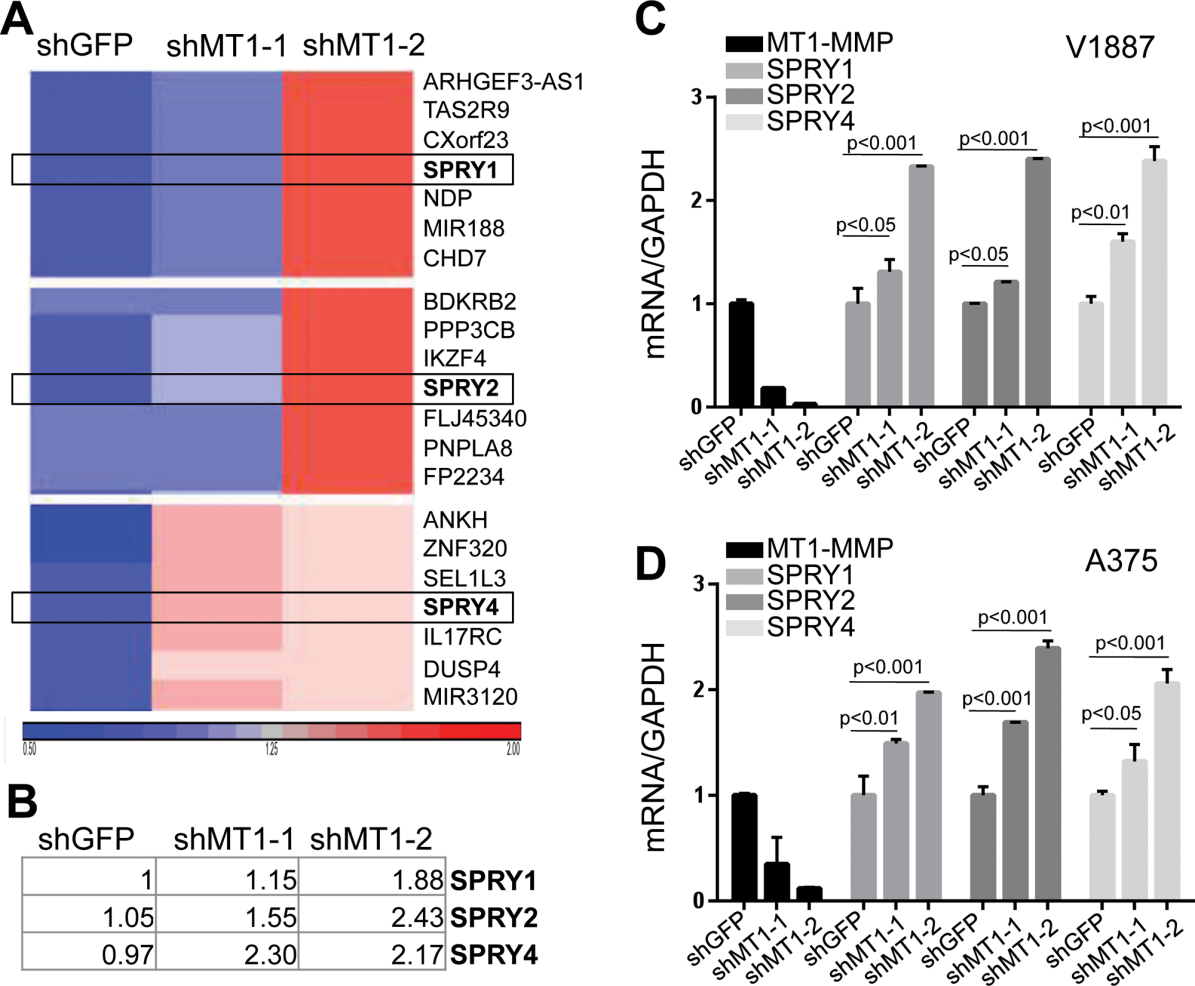
SPRY genes are novel targets of MT1-MMP **A.** HeatMap of gene expression of gene clusters containing the Sprouty genes SPRY1, 2 and 4. **B.** Fold changes of SPRY 1, 2, 4 expression (log2). SPRY1-shGFP was used as reference and arbitrarily expressed as 1. **C, D.** Expression levels of all SPRY genes in cell lines V2387 (top) and A375 (bottom) normalized to GAPDH (used as internal control). *

Given the multifaceted functions of MT1-MMP in ECM remodeling and gene modulation, we used a microarray approach to determine what genes MT1-MMP specifically regulates in melanoma. We compared the gene expression signature between 2 different shMT1-MMP expressing cells to shGFP control cells. Results show that 331 genes were differentially expressed (123 upregulated and 208 downregulated). Among the up-regulated genes, were SPRY (Sprouty) 1, 2 and 4 (Fig. 1A, 1B). These are tumor suppressors that have been shown to be inversely correlated with tumor cell growth and migration [33] and to be capable of inhibiting tumor cell motility and invasion in several cancer types [34–36]. MT1-MMP is a promoter of melanoma cell migration and metastases [14]. We therefore wanted to determine whether these SPRY genes might represent a novel mechanism through which MT1-MMP contributes to cell motility. All three SPRYs were confirmed as downstream transcriptional targets of MT1-MMP whose transcription is increased upon MT1-MMP knock down (Fig. 1C and 1D).

### MT1-MMP inhibits SPRY4 expression in malignant melanoma

**Figure 2:**
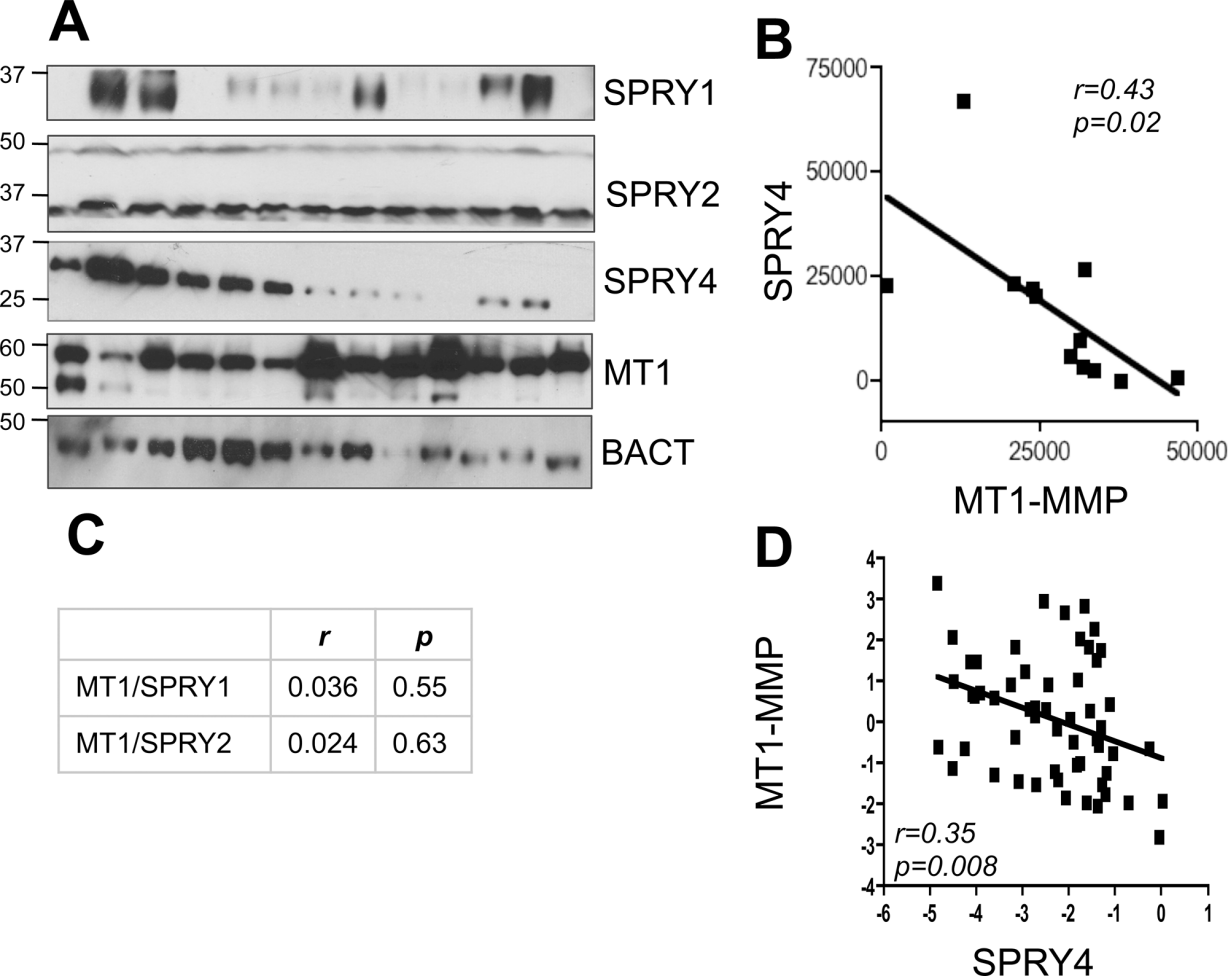
SPRY4 and MT1-MMP inversely correlate in melanoma **A.** Expression levels of MT1-MMP, SPRY1, 2 and 4 in 13 metastatic melanoma tumors. β-actin was used as loading control. **B.** correlation analysis between MT1-MMP and SPRY4 of the samples in A. Bands were quantified by ImageJ and normalized to β-actin for each sample. **C.** correlation between SPRY1 and 2 and MT1-MMP of the samples in A. **D.** Correlation between MT1-MMP and SPRY4 expression levels (mRNA) in the Riker melanoma data set (Oncomine). *n* = 56.

**Figure 3:**
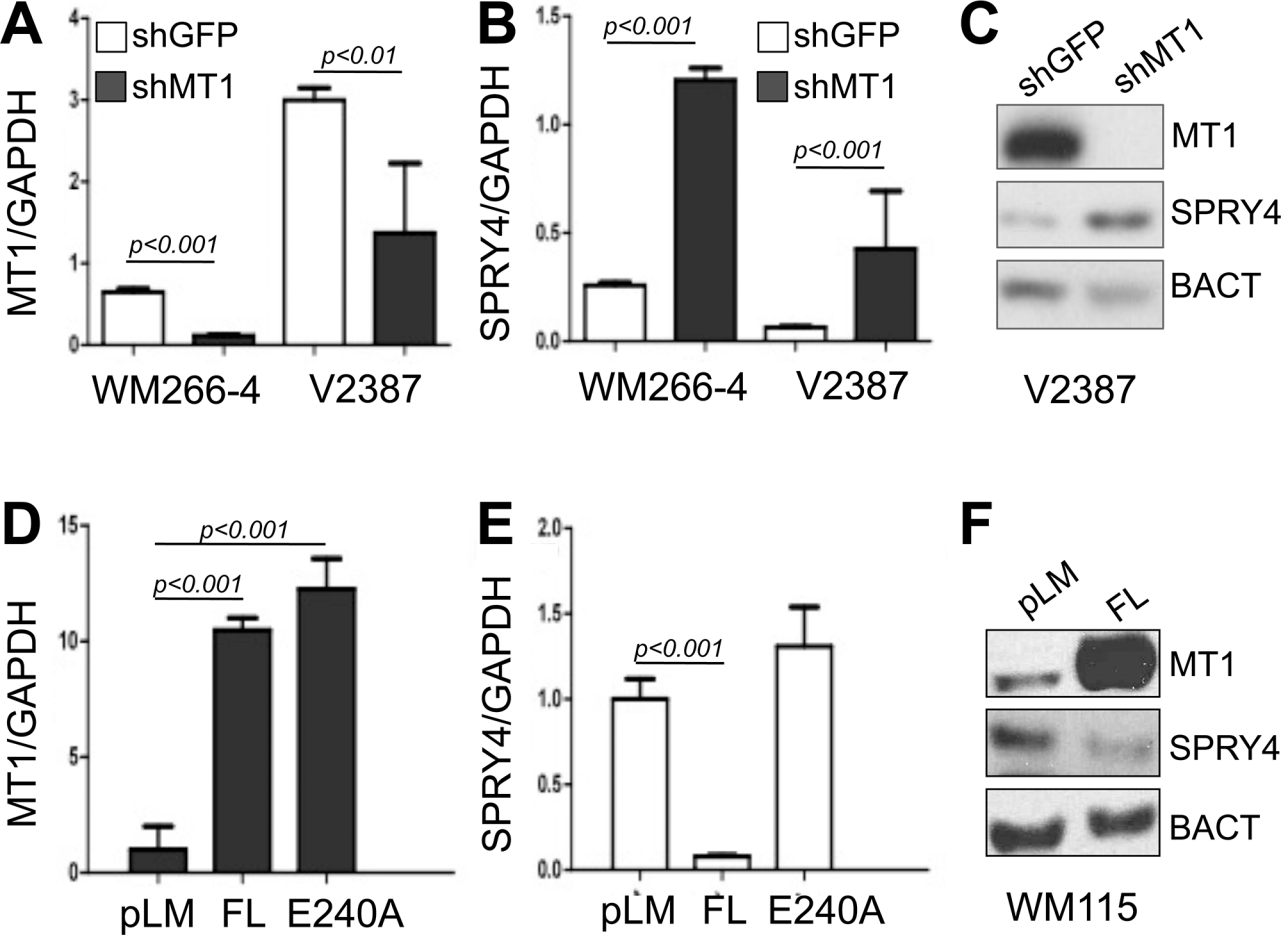
MT1-MMP inhibits SPRY4 expression **A, B.** Expression levels of MT1-MMP (A) and SPRY4 (B) in cells expressing shGFP or shMT1-MMP (shMT1–2). **C.** Protein levels of SPRY4 upon knock down of MT1-MMP in V2387 cells. **D, E.** Expression levels of MT1-MMP (both catalytically active (FL) an dead (E240A) (D)) and of SPRY4 (E) in WM115 cells. **F.** Protein levels of SPRY4 in cells expressing active MT1-MMP.

To further validate the inverse correlation between MT1-MMP and SPRY proteins and to determine whether they are expressed in melanoma, we measured the expression levels of SPRY1, SPRY2 and SPRY4 in thirteen metastatic melanoma tumor samples. All specimens expressed high levels of MT1-MMP. While SPRY2 appeared homogeneously expressed, SPRY1 and 4 expression varied among samples (Fig. 2A). However, The data in Fig. 2B and 2C reveal that only SPRY4 and MT1-MMP are significantly inversely correlated (*r* = 0.43 *p* = 0.02), whereas such negative relationship does not exist between SPRY1 and 2 and MT1-MMP (*r* = 0.036, *p* = 0.55; *r* = 0.024, *p* = 0.63, respectively). Similarly, analysis of microarray data from the Oncomine database [37] (Riker data set) also revealed an inverse correlation between MT1-MMP and SPRY4 (Fig. 2D). Based on these data we tested the functional regulation of SPRY4 by MT1-MMP. Knock down of MT1-MMP in WM266–4 and V2387 cells led again to the upregulation of SPRY4 both at the mRNA and protein levels (Fig. 3A–3C). Vice versa, overexpression of MT1-MMP in the primary cell line WM115, chosen because expresses lower levels of the protease, led to a reduction of SPRY4 mRNA and protein (Fig. 3D–3F). Interestingly, reduction of SPRY4 expression was seen only in cells expressing the catalytically active MT1-MMP, but not in those expressing the catalytically dead mutant (E240A), indicating MT1-MMP inhibits SPRY4 in a catalytically dependent manner. These data demonstrate that a functional inverse correlation exists between MT1-MMP and SPRY4 in melanoma.

### SPRY4 is inhibited in metastatic melanoma and its expression levels correlate with melanoma patient survival

**Figure 4:**
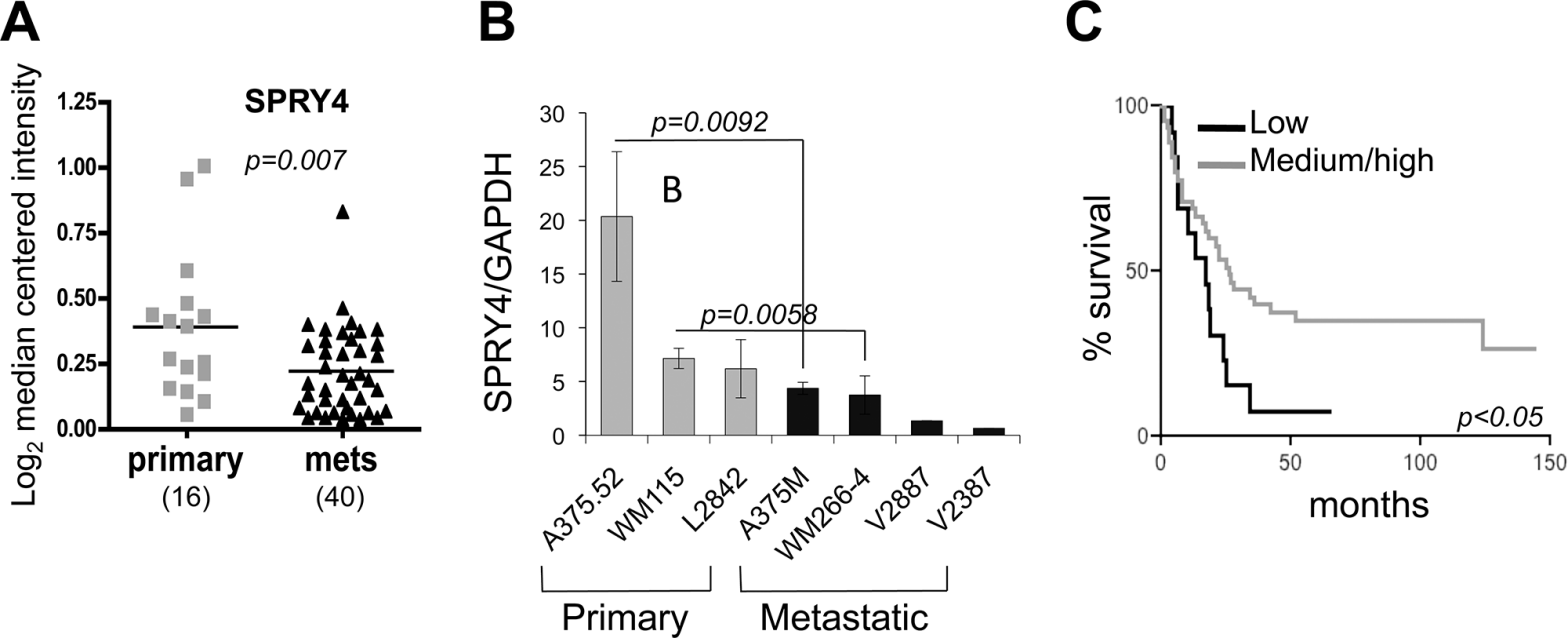
SPRY4 decreases during melanoma progression and correlates with patient outcome **A.** Expression of SPRY4 (mRNA) in the Riker data set subdivided between primary and metastatic. **B.** expression levels of SPRY4 in seven melanoma cell lines, both primary and metastatic. P values are shown for the matched syngeneic cell lines. **C.** Kaplan-Meyer survival curve indicating better survival in patients with higher SPRY4 expression.

Given that SPRY4 is a tumor suppressor that can affect cancer cell migration/invasion and based on the observed inverse correlation with MT1-MMP, we wanted to determine the association of SPRY4 with disease progression. A publicly available data set indicated a significant decrease of SPRY4 expression in metastatic samples versus primary melanoma (Riker, Fig. 4A) [37]. In contrast, unlike SPRY4, SPRY1 and 2 show a relative increase in metastatic samples (Suppl. Fig. 1). Additionally, we analyzed seven melanoma cells lines-three derived from primary melanomas and four of metastatic origin. The primary cell lines expressed higher levels of SPRY4 compared to the metastatic cell lines (Fig. 4B). Interestingly, this trend was maintained between two syngeneic pairs, the primary A375.52 and WM115 versus the metastatic A375M and WM266-4, respectively. These observations suggest that SPRY4 expression is inhibited as melanoma progresses to its metastatic form. We were not able to find a similar correlation for SPRY1 and SPRY2 (data not shown). Hence, to further assess the clinical relevance of SPRY4 in melanoma, we analyzed a melanoma tissue microarray. We used a scale of 0–3, with 0 indicating no staining, 1 low, 2 medium and 3 high staining intensity (Suppl. Fig. 2), and then grouped the samples as low (0–1) and medium-high (2–3). By comparing these scores with the patient survival data, we found that a higher expression level of SPRY4 was correlated with longer survival of melanoma patients (Fig. 4C). These data suggest that SPRY4 functions as a tumor suppressor in melanoma and its inhibition correlates with melanoma patients' poorer outcome.

### SPRY4 inhibits melanoma cell migration

**Figure 5:**
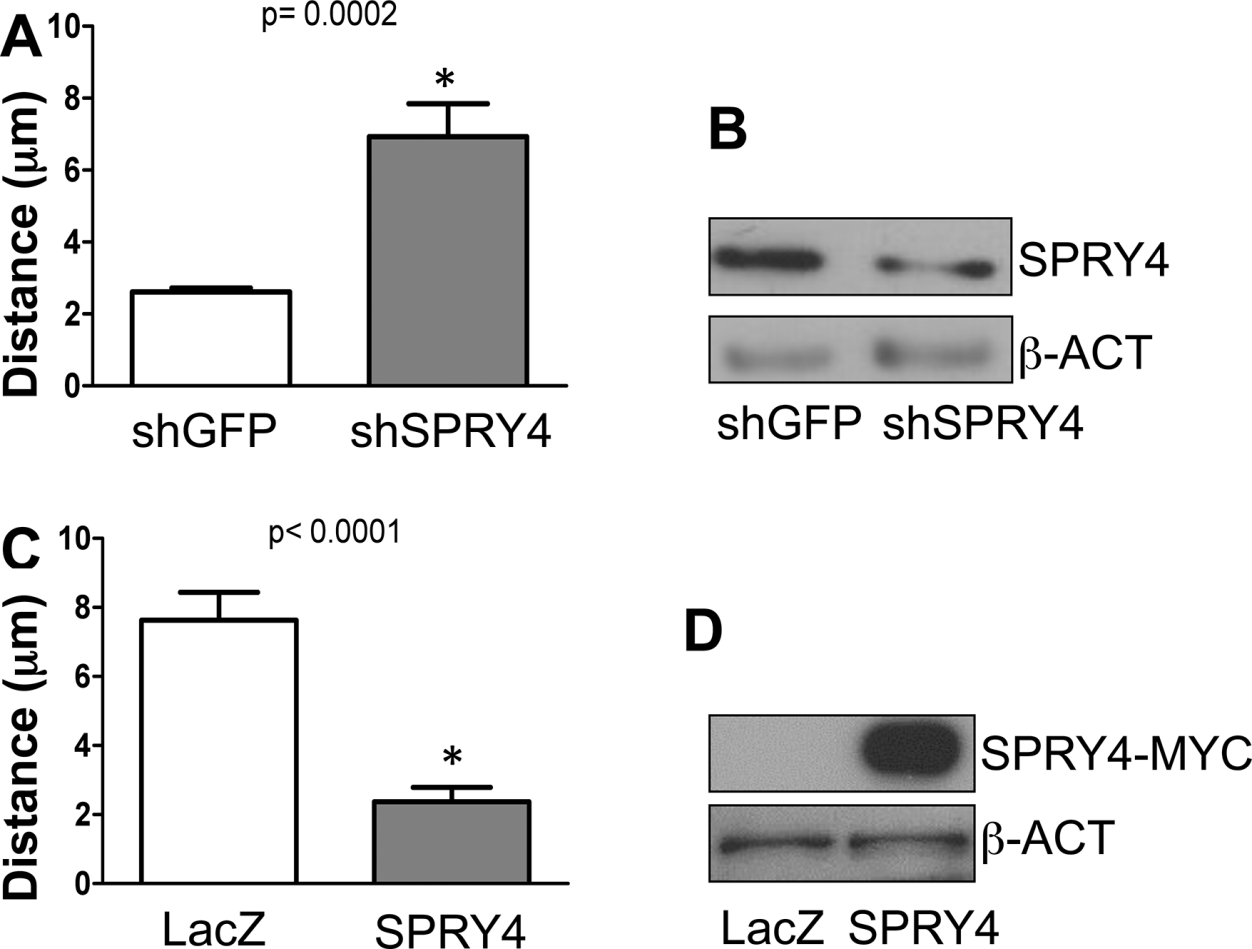
SPRY4 inhibits melanoma cell migration **A.** Relative distance travelled of WM115 cells expressing shGFP, or shSPRY4 over a 24 hours incubation period assayed by time-lapse microscopy. 30 cells were tracked per treatment. **B.** Expression of SPRY4 in shGFP and shSPRY4 cells. **C.** Relative distance travelled of WM115 cells expressing control vector (LacZ) or SPRY4 (SPRY4-Myc) over a 24 hours incubation period assayed by time-lapse microscopy. 30 cells were tracked per treatment. **D.** Expression of the Myc-tagged SPRY4 construct.

We have previously demonstrated that MT1-MMP promotes melanoma cell migration in a catalytically dependent manner [14]. SPRY proteins, including SPRY4, have been shown to inhibit cancer cell migration [34–36]. We therefore sought to determine whether SPRY4 was involved in melanoma cell migration. SPRY4 was knock down by a specific shRNA in WM115 cells (Fig. 5B). Then migration was assessed by analyzing single cell motility by time lapse microscopy as previously described [14]. We found that inhibition of SPRY4 increased melanoma cell migration, as indicated by the increase in cell distance (Fig. 5A). Conversely, overexpression of SPRY4 by an adenoviral transduction system (Fig. 5D) reduced significantly cell migration compared to LacZ controls (Fig. 5C). These data further demonstrate that SPRY4 behaves as a tumor suppressor in melanoma cells and may explain the tendency to lose SPRY4 expression as the cancer progresses.

### SPRY4 inhibits MT1-MMP mediated melanoma cell migration

**Figure 6:**
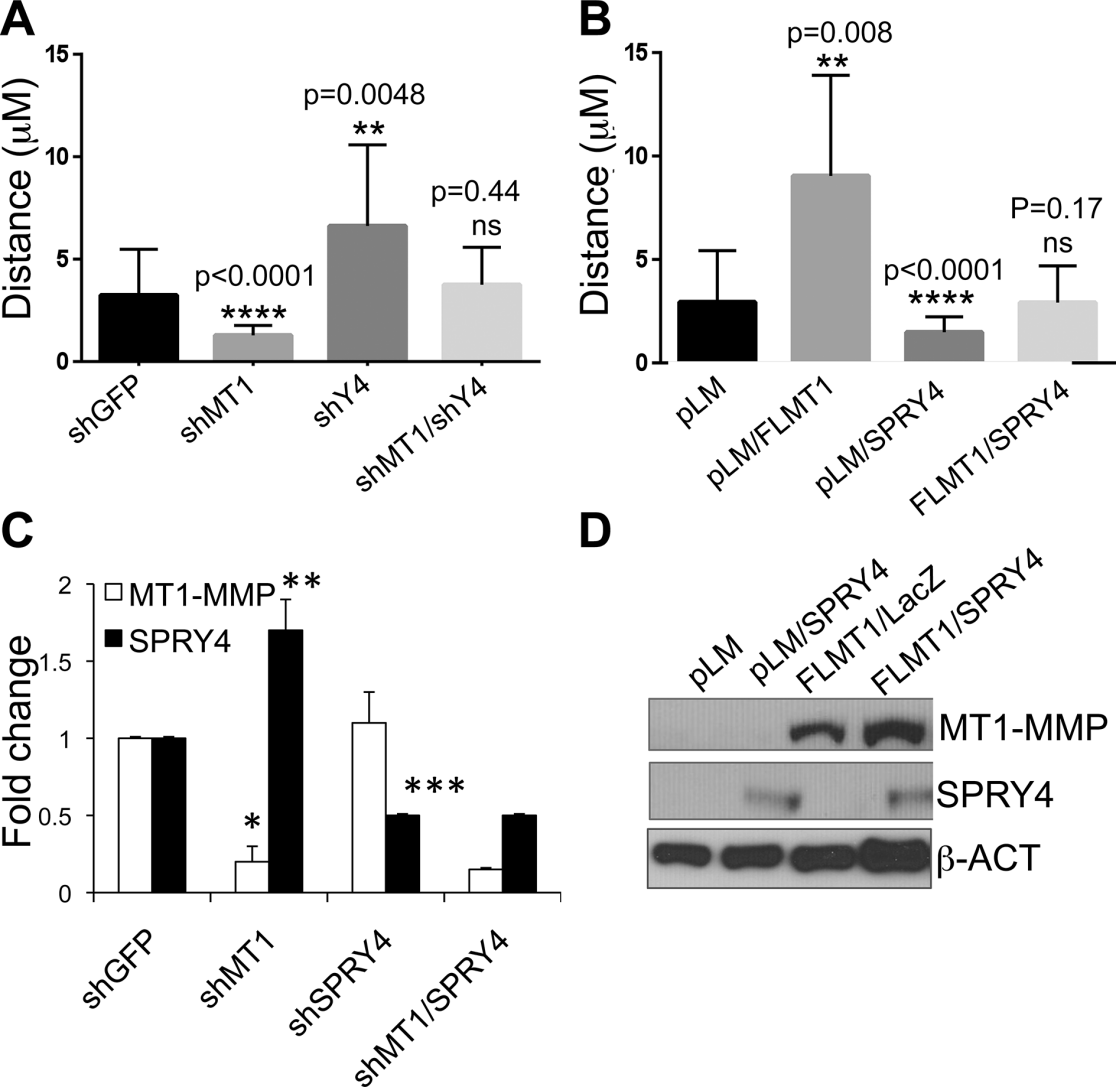
SPRY4 counteracts MT1-MMP dependent cell migration **A.** Relative distance travelled of cells expressing shGFP, shMT1-MMP, shSPRY4 alone or co-expressing both shMT1-MMP and shSPRY4, over a 24 hours incubation period assayed by time-lapse microscopy. 30 cells were tracked per treatment. **B.** Relative distance travelled of cells expressing empty vector (pLM), active MT1-MMP (FLMT1), SPRY4 or a combination of the two. **C.** Expression levels (mRNA) of MT1-MMP and SPRY4 of the cells in A. *p < 0.001_(MT1-MMP)_; **p < 0.0001_(SPRY4)_; ***p < 0.01 _(SPRY4 in shSPRY4)_ **D.** expression levels of MT1-MMP and SPRY4 of the cells in B.

Our current and published data [14] demonstrate opposite roles of MT1-MMP and SPRY4 in melanoma progression. Given that MT1-MMP negatively regulates SPRY4 expression, we next wanted to determine whether one of the mechanisms of melanoma metastasis could be the inhibition of tumor suppressors such as SPRY4 by MT1-MMP. Therefore, we co-inhibited MT1-MMP and SPRY4 in the same cells (Fig. 6C) and evaluated cell migration as previously described. Inhibition of MT1-MMP alone, reduced cell migration, while downregulation of SPRY4 increased it as expected (Fig. 6A). However, co-inhibition of both factors brought cell motility back to the level of migration of control cells (shGFP). On the other hand, overexpression of SPRY4 in cells expressing an active MT1-MMP (Fig. 6D) abrogated the migratory gain associated with overexpressed MT1-MMP (Fig. 6B). These results suggest that MT1-MMP promotes melanoma cell motility in part by inhibiting SPRY4 expression.

### MT1-MMP inhibits SPRY4 expression via MMP2/RAC1

**Figure 7:**
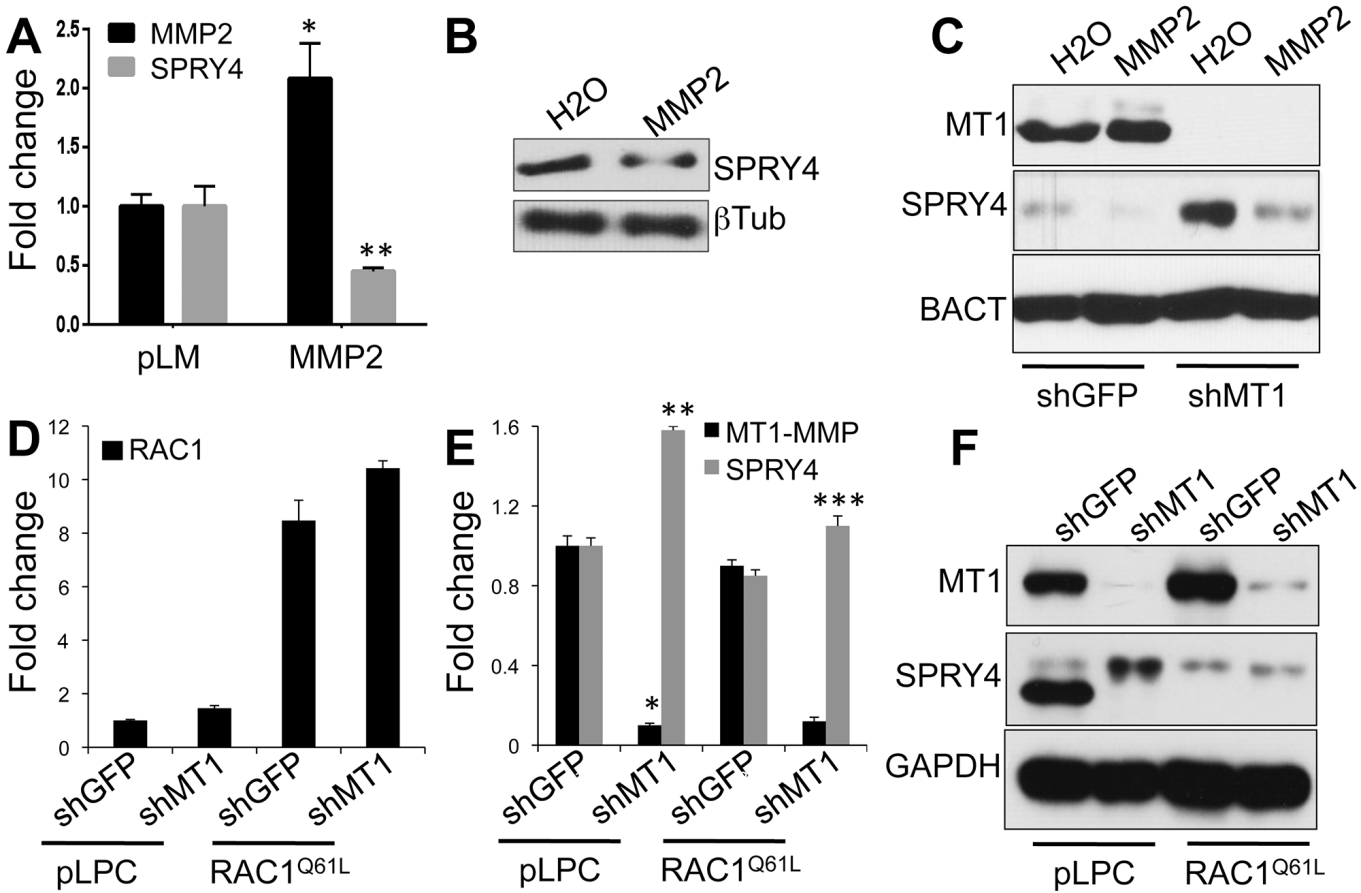
MT1-MMP modulates SPRY4 through MMP2/RAC1 **A.** Expression levels of MMP2 and SPRY4 in WM115 cells expressing a full length MMP2 construct. Values are normalized to GAPDH. *p < 0.0001; **p < 0.001. **B.** Expression of SPRY4 protein in cells treated with recombinant active MMP2 (50 ng/ml O/N). **C.** Expression of MT1-MMP and SPRY4 in cells expressing shGFP or shMT1-MMP and treated with recMMP2. An empty lane between shGFP and shMT1 samples has been cut out from the blot and lanes rejoined. **D.** Expression levels of the constitutive active RAC1 construct (RAC1^Q61L^) in cells expressing shGFP and shMT1-MMP. **E.** Expression of MT1-MMP and SPRY4 mRNAs in the cells in D. *p < 0.0001; **p < 0.0001; ***p < 0.001 (SPRY4 in RAC1^Q61L^/shMT1 vs pLPC/shMT1). **F.** Expression of MT1-MMP and SPRY4 proteins in the cells in E.

Given that the modulation of SPRY4 expression by MT1-MMP requires the catalytic activity of the protease, we speculated that an MT1-MMP substrate might be involved in SPRY4 regulation. We have previously shown that MT1-MMP mediated melanoma cell migration depends on the activation of an MMP2/RAC1 signaling axis [14]. To determine if SPRY4 lays downstream of such pathway, MMP2 was overexpressed in WM115 cells (Fig. 7A). An increase in MMP2 resulted in a decrease in SPRY4, both at the mRNA and protein levels (Fig. 7A, 7B). Importantly, expression of active recombinant MMP2 in MT1-MMP knock down cells prevented the increase in SPRY4 normally observed upon MT1-MMP inhibition (Fig. 7C). Furthermore, the expression of a constitutive active RAC1 construct (RAC1^Q61L^) (Fig. 7D) in cells expressing an shRNA against MT1-MMP, also prevented SPRY4 increase (Fig. 7E, 7F). These results suggest MT1-MMP downregulates SPRY4 expression through an MMP2/RAC1 pathway we previously demonstrated plays a critical role in mediating MT1-MMP dependent cell motility in melanoma.

## DISCUSSION

In this study, we identified SPRY4 as a novel target of MT1-MMP, a melanoma metastasis driver gene. Our results show that SPRY4 acts as a tumor suppressor in melanoma development, as its expression is inhibited in metastatic melanoma versus primary melanoma. Furthermore, high levels of SPRY4 expression predict better outcome and survival in melanoma patients.

Mechanistically, we show that SPRY4 is negatively regulated by MT1-MMP via an MMP2/RAC1 pathway, and importantly, negatively modulates MT1-MMP dependent cell migration in melanoma cells. Hence, we propose that MT1-MMP, in addition to promoting melanoma metastases by processing the ECM, does so also by repressing SPRY4, which act as a tumor suppressor in melanoma progression in part by inhibiting cell migration.

There are three mouse and four human SPRY genes with different tissue expression characteristics [38]. They play roles in the regulation of angiogenesis as well as limb, lung and kidney embryonic development [38, 39]. SPRY proteins have been shown to cross-talk with multiple pathways related to RTK signaling. For example, FGF, EGF and PDGF signaling pathways can induce SPRY4. On the other hand, SPRY4 itself can inhibit FGF induced MAPKinase activation [40] providing a negative feedback regulation of this signaling cascade. Because of these inhibitory functions in RTK signaling, SPRY proteins act as tumor suppressors in a number of cancers including breast and prostate cancer and hepatocellular carcinoma [25–27] and have been shown to regulate tumor metastatic properties by inhibiting growth receptor signaling pathways [20]. For example, SPRY4 has been shown to inhibit integrin-mediated cell spreading by interacting with testicular protein kinase 1 in prostate cancer cell lines. *In vivo* studies have shown that overexpression of SPRY4 can cause severe pulmonary hypoplasia, and loss of SPRY4 causes activation of Erk signaling in response to FGF [27, 41]. Beyond these examples the regulation of SPRY4 expression is still largely unknown.

In the present study, we show that SPRY4 expression is significantly down regulated in a majority of melanoma tumor samples and cell lines. We also examined SPRY4 mRNA expression in primary and metastatic melanoma tissue samples, obtained from the Oncomine database, and found that SPRY4 expression is decreased in metastasis compared to primary melanoma, which further suggests that SPRY4 inhibition is associated with melanoma metastasis development. Few other studies have showed SPRY4 tumor suppressor function [42]. For instance SPRY4 can inhibit proliferation and migration of NSCLC-derived cells [36]. In this study, using microarray analysis we identified SPRY4 as a novel target of MT1-MMP. SPRY1 and 2 were also identified as possible MT1-MMP targets, however, neither SPRY1 nor 2 showed any significant correlation with melanoma progression and importantly, did not correlate with MT1-MMP expression in either melanoma cell lines or tumor samples. To the best of our knowledge the present study is the first to provide evidence suggesting that SPRY4 acts as a tumor suppressor in melanoma, and it is negatively regulated by the tumor promoter proteinase MT1-MMP.

## MATERIALS AND METHODS

### Cell lines and tumor lysates

The primary melanoma cells lines WM115, A375.52, the syngeneic metastatic WM266–4, A375M and the metastatic melanoma cell line V2387 were originally purchased from ATCC or a gift or Dr. Marianne Broome Powell (Stanford University, Stanford, CA). Cells were maintained in DMEM supplemented with 10% FCS, 1% glutamine, and 1% penicillin-streptomycin. Thirteen protein tumor lysates are part of the Interdisciplinary Melanoma Cooperative Group (IMCG) collection at New York University (collected under IRB protocol #10362).

### shRNAs and expression plasmids

shRNAs against human MT1-MMP (TRCN0000 050855 and TRCN0000050856) and MMP2 (TRCN000 0051526) were purchased from Sigma. The Catalytically active MT1-MMP was a gift of Dr. Motoharu Seiki (University of Tokyo, Japan) while the MT1-MMP^E240A^ mutant (catalytically inactive) was kindly provided by Dr. Alex Strongin (Sanford Burnham, La Jolla, CA) and were previously described [14]. shRNAs against SPRY4 and SPRY4 overexpression Adeno virus were gifts from Dr. Robert Friesel (University of Maine, Orono, Maine). The constitutive active RAC1^Q61L^ construct was previously described [43].

### Western blot analysis

cell seeding, collection of protein and western blot methods were as previously described [44]. Membranes were probed with the following antibodies: anti-MT1-MMP (EMD Millipore, MA); anti–MMP2 (Santa Cruz biotechnology, CA), SPRY1, SPRY2, SPRY4 (Santa Cruz biotechnology, CA), β-actin (Santa Cruz Biotechnology, CA).

### Time-lapse video microscopy

cells were cultured on 8-well chamber slides. During the 24 hours time-lapse recording, cells were kept at 37°C/5% CO_2_. Serial phase-contrast images (10X objective) were captured at 10 minutes intervals. The images were built into a movie using the MetaMorph software. 10 cells per field for a total of three fields per sample, were highlighted and their movement followed over a 24 hours period and distance computed using the MetaMorph software.

### Immunohistochemistry

The Human Melanoma tissue microarray was purchased from IMGENEX (Cat# INH-369) and stained with an anti SPRY4 antibody from ABGEN. We used a scale of 0–3, with 0 indicating no staining, 1 the lowest and 3 the highest staining intensity, and then grouped the samples as low (0–1) and medium-high (2–3) for the Kaplan-Meyer survival curves.

### Statistics

Statistical significance of SPRY4 expression among sample types (primary vs metastasis) in the Oncomine data sets, was calculated by the Student's *t* test. A difference in expression was considered significant for *p* < 0.05. Overall patient survival was estimated by Kaplan-Meier survival analysis, and univariate associations between low and medium/high expression of SPRY4 (with respect to median) were assessed by the log-rank test. Statistical differences between groups of data generated by quantitative real time PCR, migration assays, were also calculated by the Student's *t* test. The significance of the correlation between MT1-MMP and SPRY4 was calculated by the Pearson correlation using Prism 4 (GraphPad, Inc, La Jolla, CA, USA). A correlation was considered significant for *p* < 0.05.

### Gene array

MT1-MMP was inhibited in the metastatic melanoma cell line V2387 by two different shRNAs. shGFP was used as control. Quality control (QC) reports were generated and informed us as to the usability of a sample. The “positive vs negative AUC (Area under the curve) “ method was used to vet the samples. Samples with values less than 0.8 (an Affymetrix guidline) were dismissed from the study. At that point, expression signals were regenerated using the Robust Multichip Analysis (RMA) and only the QC-passing samples. An expression change ratio cutoff was set at 1.5 or greater. For the clustering in Fig. 1A the gene expression matrix containing measured gene expression values for each gene under each condition (shGFP, shMT1–1, shMT1–2) was row-normalized by dividing the value for each gene by the mean of expression for that gene in all conditions. Heat Maps were obtained by using log2 transformed normalized data. Only values with a standard deviation > 0.5 were considered.

### Real-time PCR

cDNA was synthesized from total RNA using SuperScript first-strand synthesis system for RT-PCR (Invitrogen), then used for PCR amplification with SYBR Green PCR master mix (Roche). The following primer sets were used to amplify specific target genes: human GAPDH forward: 5′-CGCTCTCTGCTCCTCCTGTT-3′; reverse: 5′-CCATGGTGTCTGAGCGATGT-3′; human MT1-MMP forward: 5′-CTCCCTCGGCTCGGCCCAAA-3′; reverse: 5′-CGCCTCATGGCCTTCATGGTGTCT-3′; human MMP2: forward: 5′-TGATCTTGACCAGAATACCAT TGA-3′; reverse: 5′-GGCTTGCGAGGGAAGAAGTT-3′;human SPRY1: forward: 5′-AGGGCTATCTTCCT AGCA-3′; reverse: 5′-GTGAGAAGCATGGGGT-3′; human SPRY2: forward: 5′GCGATCACGGAGTTCAG-3′; reverse: 5′-GTGGAGTCTCTCGTGT-3′; human SPRY4: forward:5′-CCAGGATGTCACCCACCATTG-3′; reverse: 5′-TGTGCTGCTGCTGCTC-3”. Relative quantification of mRNA expression levels was normalized by GAPDH.

## SUPPLEMENTARY FIGURES


