# Supplementary material for: MT1-MMP dependent repression of the tumor suppressor SPRY4 contributes to MT1-MMP driven melanoma cell motility
Source: Oncotarget. 2015 Sep 12;6(32):33512–22. doi: 10.18632/oncotarget.5258 (PMC4741782; doi:10.18632/oncotarget.5258)
Supplement: Supplementary file 1 [file oncotarget-06-33512-s001.pdf]

SUPPLEMENTARY FIGURES

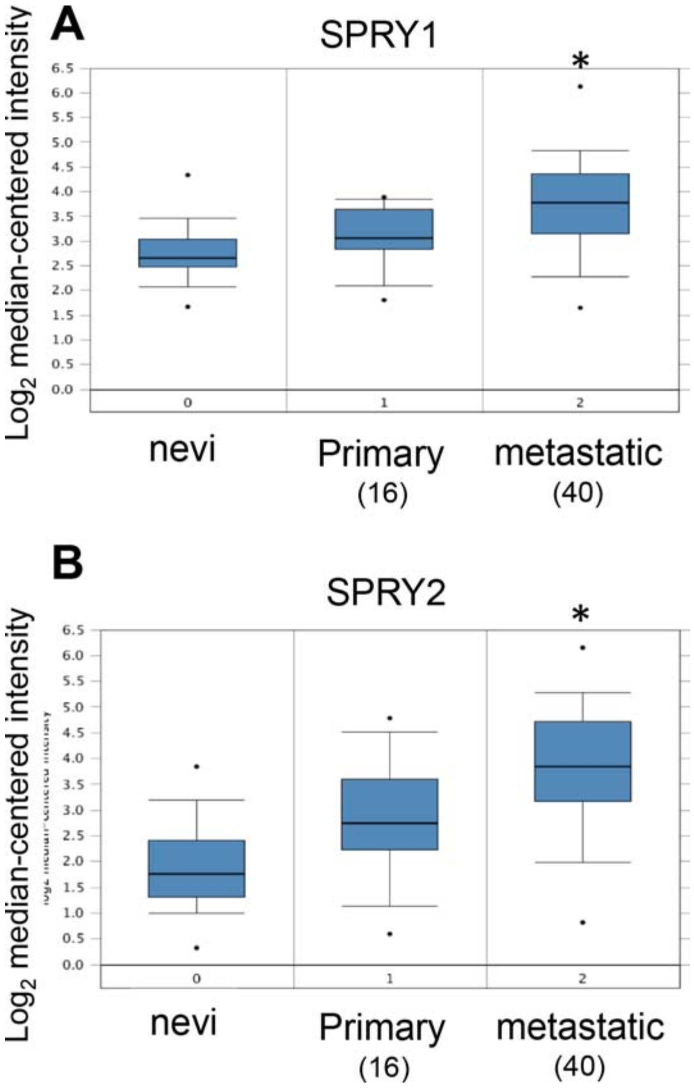

Supplementary Figure S1: SPRY1 and SPRY2 are increased in metastatic melanoma: Riker data set. Expression levels of both SPRY1 and 2 are increased in metastatic melanoma samples versus primary lesions. *$p < 0.05$.

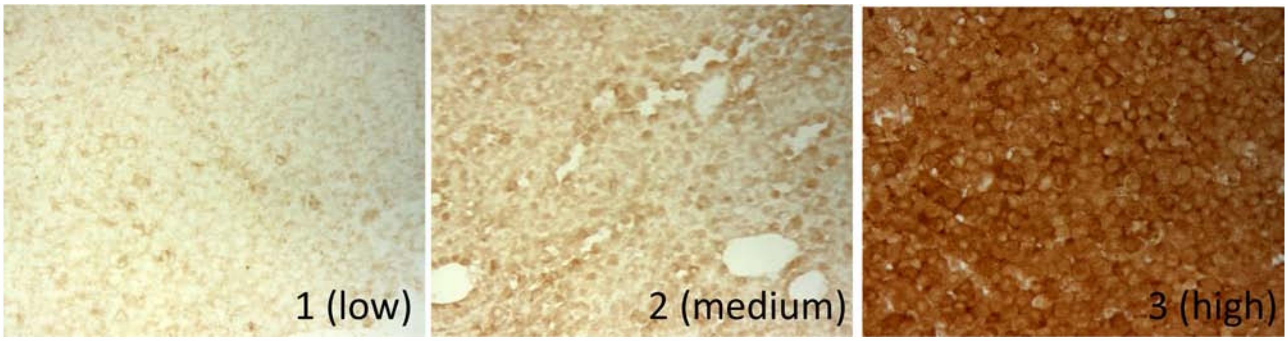

Supplementary Figure S2: staining intensity of the tissue array.
